# Implementation of a Pan-Genomic Approach to Investigate Holobiont-Infecting Microbe Interaction: A Case Report of a Leukemic Patient with Invasive Mucormycosis

**DOI:** 10.1371/journal.pone.0139851

**Published:** 2015-11-10

**Authors:** Samuel A. Shelburne, Nadim J. Ajami, Marcus C. Chibucos, Hannah C. Beird, Jeffrey Tarrand, Jessica Galloway-Peña, Nathan Albert, Roy F. Chemaly, Shashank S. Ghantoji, Lisa Marsh, Naveen Pemmaraju, Michael Andreeff, Elizabeth J. Shpall, Jennifer A. Wargo, Katayoun Rezvani, Amin Alousi, Vincent M. Bruno, Phillip A. Futreal, Joseph F. Petrosino, Dimitrios P. Kontoyiannis

**Affiliations:** 1 Department of Infectious Diseases, University of Texas MD Anderson Cancer Center, Houston, Texas, United States of America; 2 Department of Genomic Medicine, University of Texas MD Anderson Cancer Center, Houston, Texas, United States of America; 3 The Alkek Center for Metagenomics and Microbiome Research and the Department of Molecular Virology and Microbiology, Baylor College of Medicine, Houston, Texas, United States of America; 4 Department of Microbiology & Immunology and Institute for Genome Sciences, University of Maryland School of Medicine, Baltimore, Maryland, United States of America; 5 Department of Laboratory Medicine, University of Texas MD Anderson Cancer Center, Houston, Texas, United States of America; 6 Department of Leukemia, University of Texas MD Anderson Cancer Center, Houston, Texas, United States of America; 7 Department of Stem Cell Transplantation and Cellular Therapy, University of Texas MD Anderson Cancer Center, Houston, Texas, United States of America; 8 Department of Surgical Oncology, University of Texas MD Anderson Cancer Center, Houston, Texas, United States of America; CAS-MPG PICB, CHINA

## Abstract

Disease can be conceptualized as the result of interactions between infecting microbe and holobiont, the combination of a host and its microbial communities. It is likely that genomic variation in the host, infecting microbe, and commensal microbiota are key determinants of infectious disease clinical outcomes. However, until recently, simultaneous, multiomic investigation of infecting microbe and holobiont components has rarely been explored. Herein, we characterized the infecting microbe, host, micro- and mycobiomes leading up to infection onset in a leukemia patient that developed invasive mucormycosis. We discovered that the patient was infected with a strain of the recently described *Mucor velutinosus* species which we determined was hypervirulent in a *Drosophila* challenge model and has a predisposition for skin dissemination. After completing the infecting *M*. *velutinosus* genome and genomes from four other *Mucor* species, comparative pathogenomics was performed and assisted in identifying 66 *M*. *velutinosus*-specific putatively secreted proteins, including multiple novel secreted aspartyl proteinases which may contribute to the unique clinical presentation of skin dissemination. Whole exome sequencing of the patient revealed multiple non-synonymous polymorphisms in genes critical to control of fungal proliferation, such as *TLR6* and *PTX3*. Moreover, the patient had a non-synonymous polymorphism in the *NOD2* gene and a missense mutation in *FUT2*, which have been linked to microbial dysbiosis and microbiome diversity maintenance during physiologic stress, respectively. In concert with host genetic polymorphism data, the micro- and mycobiome analyses revealed that the infection developed amid a dysbiotic microbiome with low α-diversity, dominated by staphylococci. Additionally, longitudinal mycobiome data showed that *M*. *velutinosus* DNA was detectable in oral samples preceding disease onset. Our genome-level study of the host-infecting microbe-commensal triad extends the concept of personalized genomic medicine to the holobiont-infecting microbe interface thereby offering novel opportunities for using synergistic genetic methods to increase understanding of infectious diseases pathogenesis and clinical outcomes.

## Introduction

**Fig 1:**
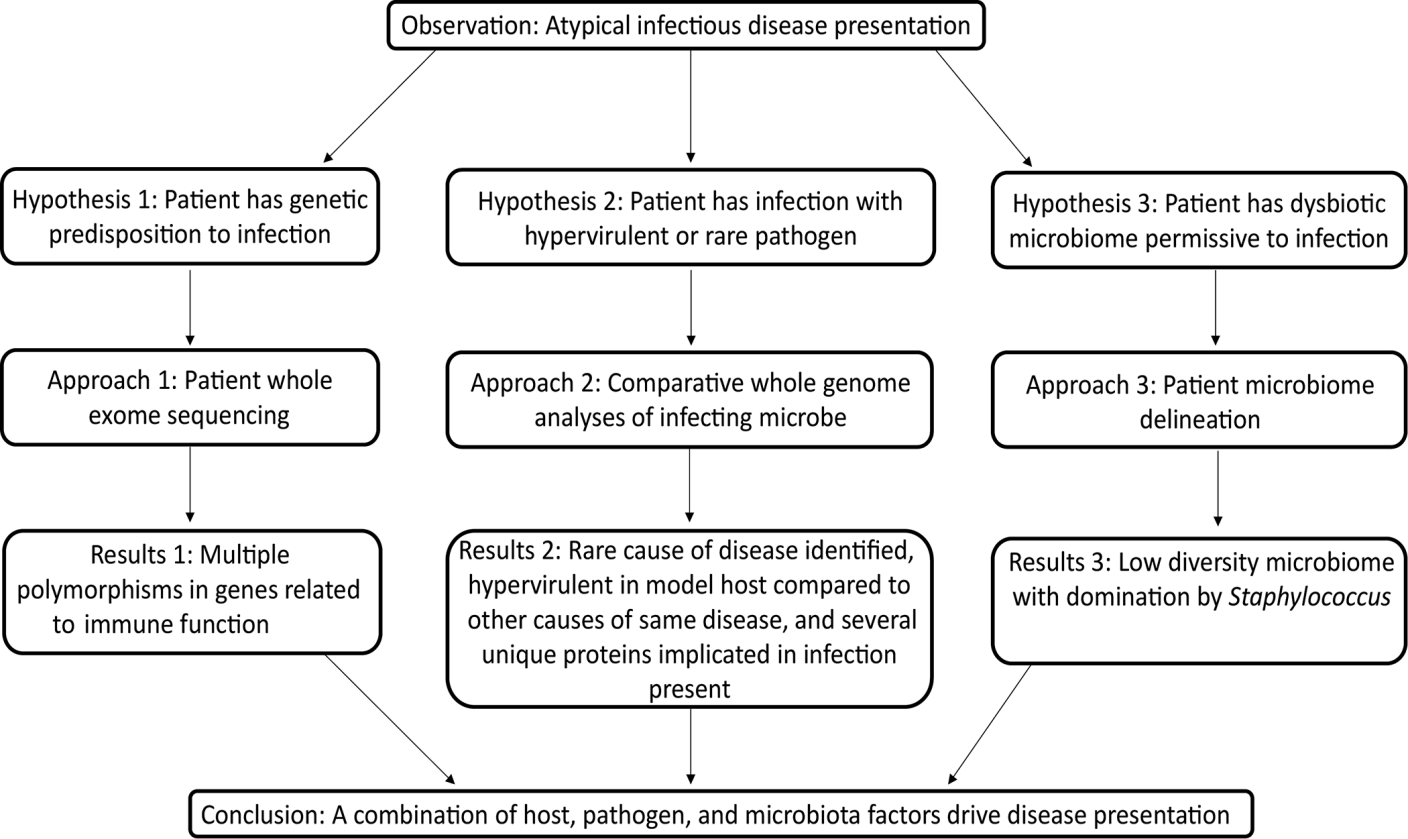
Summary of hypotheses, methods, and results for this case study.

With the rapidly emerging understanding of the critical role played by microbiota in human health and disease, it is becoming increasingly important to consider the human as holobiont, which entails the concept of the host in conjunction with its commensal microorganisms [1]. In this context, the occurrence and clinical course of an infectious disease is the result of interactions between the infecting microbe, host, and commensal microorganisms, the outcome of which has been called the damage-response framework of microbial pathogenesis [1]. To date, the overwhelming majority of infectious disease research has focused on a single aspect of the infecting microbe-host-commensal microorganism paradigm, such as studying pathogen evolution via large scale sequencing of bacterial isolates [2], investigating the role of host genetic polymorphisms in infectious diseases susceptibility [3], or characterizing how the intestinal microbiome affects infection predisposition [4]. However, it is likely that advancing understanding of human infections will be optimized only when multiple facets of the holobiont-infecting microbe interaction are studied simultaneously [1, 5]. For example, it was recently shown in the case of rheumatoid arthritis that a network of multi*omics* approaches, host-microbiome interface data, and computational intensive systems biology can be used to describe the complexity of disease, provide a platform for *in silico* hypothesis testing, and determine the possible adverse impact of therapies [6]. Herein, we demonstrate the power of implementing a comprehensive host-pathogen-microbiome genomic framework approach in order to gain a deeper understanding of an individual patient's disease. Specifically, we combined genetic analyses of the infecting agent, host whole exome sequencing, and longitudinal determination of the oral and stool micro- and mycobiomes leading up to disease onset to explore the atypical disease presentation of a leukemic patient with invasive mucormycosis (Fig 1).

## Case Report

**Fig 2:**
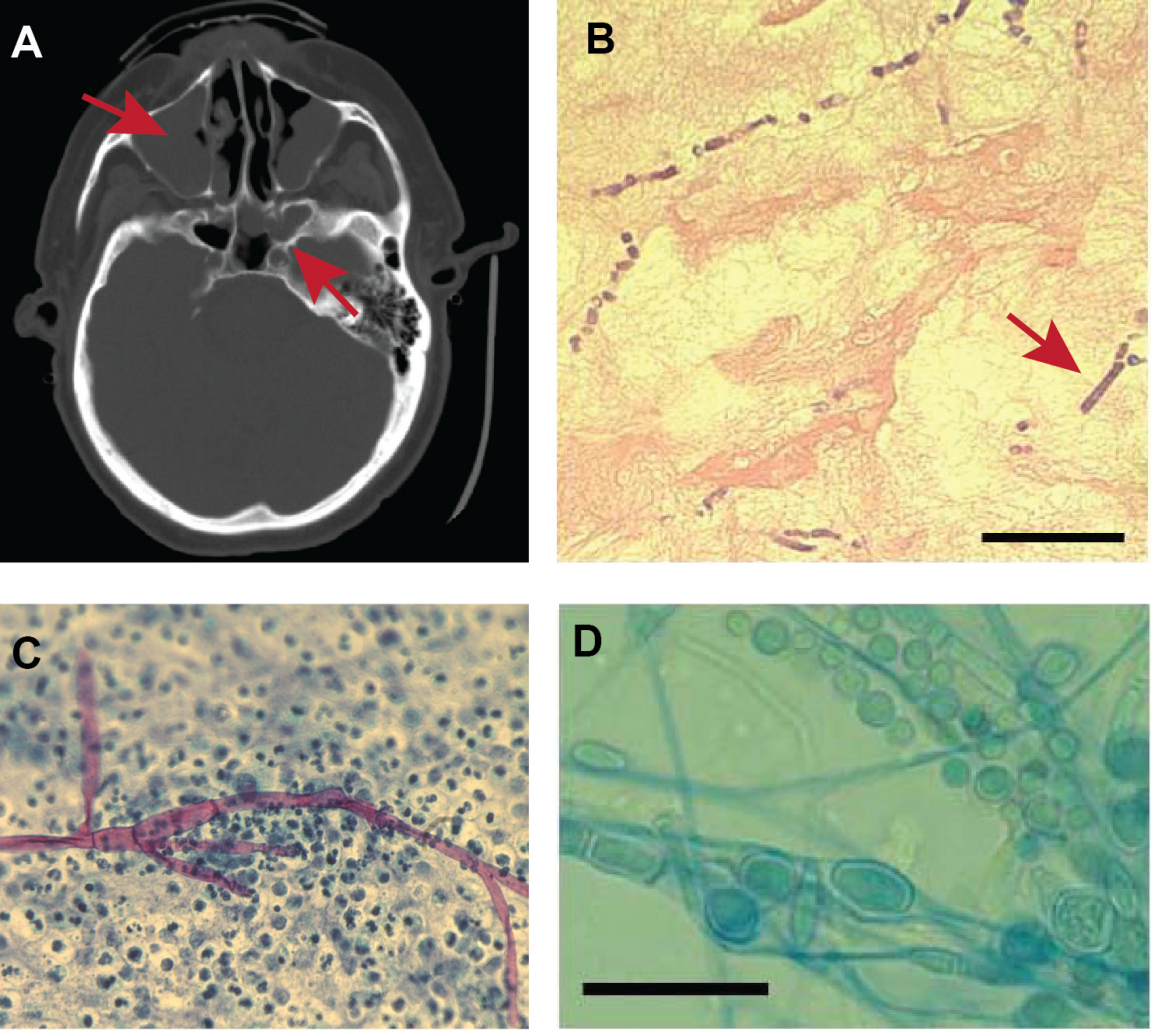
Clinicopathologic description of invasive mucormycosis and RM1 strain characterization. (A) Computerized tomography showing multi-focal sinusitis (red arrows). (B) 20x magnification of hematoxylin and eosin stain of nasal tissue showing chaining chlamydospores in an area of necrotic tissue mimicking toruloid septate hyphae (red arrow shows immature chlamydospores). Scale bar = 50 μM. (C) 20x magnification of Papanicolaou stain of biopsy of lower extremity nodule showing chronic inflammation and invasive, non-septated hyphal form. (D) 100x magnification of microscopic morphology of strain RM1grown on Sabourad-Dextrose-Emmons agar at 30°C for 48 hrs showing barrel-shaped chlamydospores that resemble pauci-septate hyphae. Scale bar = 10 μM.

A 68 year old man with a history of recalcitrant onychomycosis was treated for acute myelogenous leukemia (AML) with vosaroxin and decitabine, and he developed severe, persistent neutropenia (neutrophils < 100/μl) on day 10 of therapy for which he received prophylactic ciprofloxacin, voriconazole, and valacyclovir. Unexplained fevers began on day 24, and a CT scan showed invasive sinusitis (Fig 2A). Nasal biopsy revealed tissue invasion by septated hyphae consistent with hyalohyphomycetes (Fig 2B). However, the clinical lab reported the culture of the sinus tissue grew a *Rhizomucor spp*. The patient was treated with liposomal amphotericin B (5 mg/kg/day) and posaconazole tablets (300 mg/day) with steady state serum posaconazole levels averaging 1600 ng/mL. Liposomal amphotericin B was stopped after two months. Three weeks later the patient presented with new neutropenic fever and a nodular skin lesion in his leg. Biopsy showed invasive hyphal elements consistent with disseminated mucormycosis (Fig 2C). The patient was restarted on liposomal amphotericin B for an additional five months with gradual improvement.

## Materials and Methods

### Details of Sampling

Ten patients, to include the case patient, were enrolled in a longitudinal, observational study of the gastrointestinal microbiome occurring during the course of induction chemotherapy for newly diagnosed AML. The study was approved by the MD Anderson Cancer Center IRB (PA13-0339), and written informed consent was obtained. From a micro- and mycobiome standpoint, swabs of the buccal mucosa and stool samples were obtained immediately prior to initiating induction chemotherapy. Subsequent sampling occurred bi-weekly and was stopped when neutrophils returned to greater than 500 per μL. Buccal swabs and stool samples were stored at -80°C until processed.

### Fungal Strain Characterization and Sequencing

The strain identified by the clinical laboratory to be a *Rhizomucor spp*. (named RM1) was isolated from the nasal surgical specimen. Morphologic characterization of RM1 was performed following growth on freshly prepared Sabouraud dextrose agar plates. Anti-fungal susceptibility testing was performed following Clinical Laboratory Standard Institute (CLSI) guidelines (Reference Method for Broth Dilution Antifungal Susceptibility Testing of Filamentous Fungi; Approved Standard). Fungal DNA was prepared from 30 mg of freeze-dried mycelium that was ground into a fine powder as previously described [7]. Gene specific sequences from RM1 were obtained using PCR followed by Sanger sequencing using previously published primer sets [8, 9] and compared to sequences obtained from NCBI. Following alignment using CLUSTALW, sequences were analyzed in MEGA version 6.1 to create radial trees using the neighbor-joining statistical method and the maximum- likelihood composite model [10]. The robustness of the nodes was evaluated via bootstrapping (1000 replicates). Whole genome sequencing of RM1 was performed using 250-bp paired end reads on an Illumina MiSeq following the manufacturer’s instructions. A total of 500 megabases of sequence data was obtained and aligned to publicly available *Mucor* genomes and to the *Mucor* genomes determined in the course of this study using Geneious version 7.2.

Details on the *Mucor* strains for which genomes were determined during this study are as follows: *M*. *velutinosus* B5328 was obtained in 1993 from the nasal cavity of a human in South Carolina, USA. *M*. *circinelloides* B8987 is also a human isolate collected in Georgia, USA in 2010. *M*. *racemosus* B9645 is an environmental isolate collected from a room in Florida, USA in 2012.*M*. *indicus* B7402 was obtained from a human in Georgia, USA in 2008. The genomes of the following two *Mucor* strains were publicly available when this study began: *M*. *irregularis* B50 is a human skin isolate from Nanjing, China, and *M*. *circinelloides f*. *circinelloides* 1006 PhL is also a human skin isolate that has been previously described [11, 12].

The genome sequences of *M*. *velutinosus* B5328, *M*. *circinelloides* B8987, *M*. *indicus* B7402, and *M*. *racemosus* B9645 were generated at the Institute for Genome Sciences (IGS) Genomics Resource Center (http://www.igs.umaryland.edu) using a combination of paired-end libraries and mate-pair libraries on the Illumina HiSeq 2000. Draft genomes were assembled using the MaSuRCA v.1.9.2 genome assembler.[13] *M*. *velutinosus* B5328 had an estimated sequencing coverage of 108x and 35,967,610 bases that assembled into 1,595 scaffolds with GC% of 40.52. *M*. *circinelloides* B8987 (100.43x coverage) had 36,770,135 bases assemble into 1,582 scaffolds with GC% of 39.46. *M*. *indicus* B7402 (55.87x coverage) had 1,304 scaffolds comprising 39,917,353 bases with GC% 35.81. *M*. *racemosus* B9645 (coverage of 49.38x) had 65,656,770 bases that assembled into 5,324 scaffolds with 32.47% GC content.

Genomes assembled by IGS, as well as *M*. *irregularis* B50 scaffolds downloaded from GenBank, were annotated with the IGS Eukaryotic Annotation Pipeline protocol 1.0 at the Institute for Genome Sciences, Informatics Resource Center (http://www.igs.umaryland.edu), which incorporates repeat masking, multiple *ab initio* gene prediction algorithms, spliced protein alignments, transcript evidence when available, and an evidence combiner, as well as non-coding RNA prediction. Proteins were generated by translating the gene feature file of each *Mucor* strain using a script, resulting in the following number of proteins: 8,780 B50; 9,855 B5328; 11,935 B7402; 10,687 B8987; and 15,859 B9645. For *M*. *circinelloides* 1006PhL 12,227 proteins were downloaded from NCBI.

In order to identify putatively secreted proteins SignalP v.4.1 was run on predicted proteomes to detect the presence and location of signal peptide cleavage sites [14]. Protein families conserved among all six *Mucor* strains were detected by first predicting orthologs (genes similar between species due to common ancestry) for every taxon:taxon pair with InParanoid 4.1 [15] followed by filtering with MultiParanoid [16]. InParanoid used the two-pass BLAST strategy, the outgroup *Rhizopus delemar* 99–880, and with other parameters set to default. A multiple sequence alignment was generated for each protein group using MUSCLE [17]. Alignments were concatenated into one super alignment and gapped regions were removed with Gblocks_0.91b [18]. Phyml [19] was run on the block alignment with the following parameters: NNIs tree topology search, BioNJ initial tree, LG amino acid substitution model, 100 bootstrap replicates. The resulting tree was visualized in FigTree v1.4.2 (http://tree.bio.ed.ac.uk/software/figtree/).

The model host, *Drosophila melanogaster* (N = 22 per test group), was used for pathogen challenge experiments where all experiments were performed in triplicate on three different days at the same time in order to avoid differences in circadian rhythm as previously described [20]. The clinical isolates S1, S2, and S3 were previously confirmed to be *Mucor circinelloides* species [21].

### Patient Whole Exome Sequencing and Analysis

Human gDNA was isolated from buccal mucosa as previously described [22]. Whole exome capture with SureSelect Human All Exon V4 baits from Agilent Technologies was sequenced at 205 bp average insert size to 60X depth paired-end reads using Illumnia HiSeq 2000. Alignment and annotation were done as previously described [22]. Germline variant calls were made using Platypus [23]. Potentially significant polymorphisms were identified in 844 human genes known or putatively involved in innate immunity (http://www.innatedb.com/moleculeSearch.do) including genes previously identified as important in innate anti-fungal immunity such as C-type lectins and downstream signaling adaptor molecules, Toll-like receptors and downstream signaling molecules, and chemokine receptors [24, 25]. Polymorphisms were queried in the NHLBI exome server (http://evs.gs.washington.edu/EVS/) to determine the predicted functional significance, and those polymorphisms predicted to negatively affect protein function were then searched in the PubMed (http://www.ncbi.nlm.nih.gov/pubmed) and dbSNP (http://www.ncbi.nlm.nih.gov/SNP) databases for published data regarding the potential association of polymorphisms with disease phenotypes. Additional, correlative searches were performed in the HUGE navigator database (www.hugenavigator.net). Finally, manual inspection of all insertions, deletions, or single nucleotide polymorphisms predicted to truncate or extend open reading frames was performed to identify any potential contributions to alteration in immune function.

### Microbiome Analyses

16S rRNA gene and ITS sequencing methods were adapted from the methods developed for the NIH-Human Microbiome Project [26, 27]. Briefly, bacterial genomic DNA was extracted from stool samples and buccal swabs using MO BIO PowerSoil DNA Isolation Kit (MO BIO Laboratories). The 16S rDNA V4 region was amplified by PCR and sequenced in the Illumina MiSeq platform using the 2x250 bp paired-end protocol. For mycobiome analysis, the Internal Transcribed Spacer 2 (ITS2) region was targeted for amplification using primers ITS3 and ITS4 [28] and amplicons were sequenced in the Illumina MiSeq platform using the 2x300 bp paired-end protocol. All the primers used for amplification contained adapters for MiSeq sequencing and indexed barcodes so that the PCR products may be pooled and sequenced directly [29].

The 16S rRNA gene and ITS2 analysis pipelines incorporate phylogenetic and alignment-based approaches to maximize data resolution. The read pairs were demultiplexed based on the unique molecular barcodes, and reads were merged using USEARCH v7.0.1001 [30]. Both pipelines leverage the QIIME (Quantitative Insights Into Microbial Ecology) software package [31] developed at the University of Colorado, as well as several other tools, including custom analytic packages developed at the Baylor College of Medicine Center for Metagenomics and Microbiome Research to provide summary statistics and quality control measurements for each sequencing run, as well as multi-run reports and data-merging capabilities for validating built-in controls and characterizing microbial communities across large numbers of samples or sample groups. The resulting sequences were assigned into Operational Taxonomic Units (OTUs) at a similarity cutoff value of 97% using the UPARSE pipeline in QIIME and the SILVA (bacteria) and UNITE (fungal) databases [32, 33]. Abundances were recovered by mapping the demultiplexed reads to the UPARSE OTUs. A custom script constructed an OTU table from the output files generated in the previous two steps, and then used to calculate alpha-diversity (i.e Shannon diversity index), beta-diversity [34], and provide taxonomic summaries. Each round of microbial and fungal DNA amplification includes a known and previously sequenced control of bacterial DNA (*Francisella tularensis*) which is not expected to be found in human samples. In addition, for the ITS pool, we included a fungal mock community (composed of *Candida spp*., *Saccharomyces spp*., *Cryptococcus spp*., *Penicillium spp*., and *Clavispora spp*.) previously characterized by whole genome shotgun sequencing to evaluate the ITS analytic pipeline. Negative controls, or non-template controls, were also included and pooled at the average sample volume.

## Results

### Fungal strain characterization

**Fig 3:**
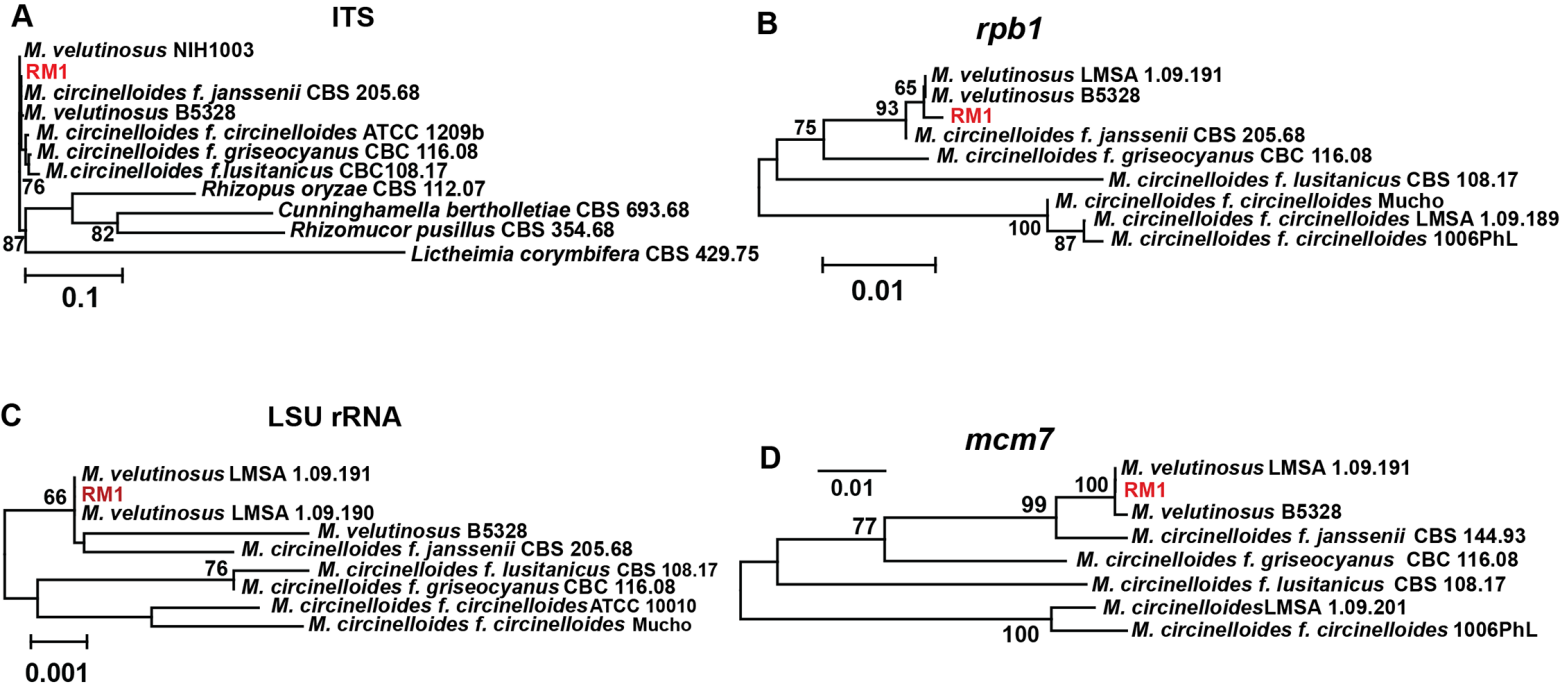
Identification RM1 as a member of the *M*. *velutinosus* species. (A-D) Genetic relatedness among various *Mucor* strains as determined by comparing sequence of indicated RM1 genes to sequences obtained from NCBI. Nucleotide sequences were aligned using MUSCLE and a neighbor-joining phylogenetic tree was created in MEGA version 6.1 using the Maximum-Likelihood method. Bootstrap iteration frequencies of >60% (1,000 iterations) are shown in the nodes. Branch lengths are proportional to genetic distance indicated by scale bar.

The vast majority of mucormycoses afflicting AML patients occur during the relapsed/refractory stage of the disease [35] and dissemination of mucormycosis to the skin is decidedly unusual, as cutaneous mucormycosis is almost exclusively due to traumatic inoculation [36]. Thus, we sought to gain insight into the unusual timing/presentation of mucormycosis in our patient through molecular characterization of the infecting agent (RM1). Using targeted gene sequencing of genes described in the Fungal Tree of Life Project [8], we identified RM1 as belonging to the recently described *M*. *velutinosus* species (Fig 3). Although the presence of septated hyphae is thought to be indicative of a non-*Mucorales* mold, similar to other *M*. *velutinosus* strains [37], RM1 produced chlamydospores that resemble hyphae with septations (Fig 2D), thereby explaining the “septated hyphae” observed in the initial surgical culture. Moreover, the posaconazole minimum inhibitory concentration for RM1 was 4,000 ng/μL, which extends previous data regarding non-susceptibility of *M*. *velutinosus* to posaconazole [38] and is consistent with the infection relapse in our patient when posaconazole was substituted for liposomal amphotericin B.

**Fig 4:**
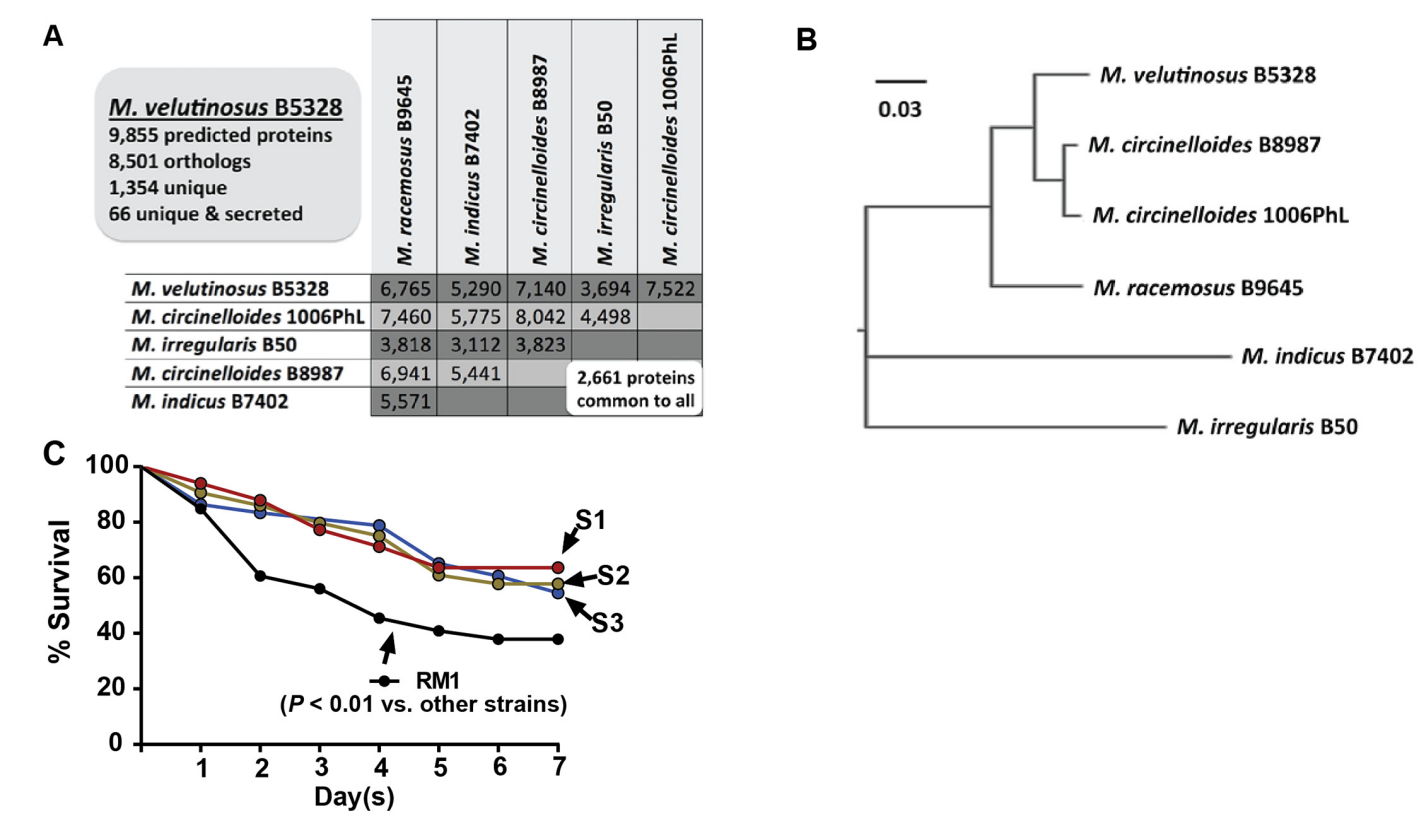
Genomic and phenotypic characterization of RM1 as a virulent strain of *M*. *velutinosus*. (A) Number of orthologs common to sequenced *Mucor* strains, with detailed information about *M*. *velutinosus* B5328 depicted in the shaded box and total number common to the six sequenced *Mucor* strains shown in the white box. (B) Midpoint-rooted tree depicting the phylogenetic relationship of six sequenced *Mucor* strains using the 2,661 proteins common to all 6 strains. Scale bar indicates genetic distance. (C) Survival curves of *Drosophila melanogaster* following challenge with indicated *Mucor* strains (n = 22 flies per challenge group) with experiment repeated in triplicate. *P* value refers to log-rank test for survival time.

There are currently only a small number of published *M*. *velutinosus* infections in humans [37–39], and, remarkably, hematogenous dissemination has been observed in nearly all cases. Thus, we sought to gain insight into the clinical scenario of hematogenous dissemination by characterizing how the RM1 genome differs from genomes of other *Mucor* strains. Two of the comparison genomes (*M*. *irregularis* B50 and *M*. *circincelloides f*. *circinelloides*1006 PhL) were publicly available whereas the other four were determined as part of this study. Greater than 99.9% of the reads from RM1 aligned to *M*. *velutinosus* strain B5328 for which basic assembly statistics are available in Table 1. Compared to genomes from 5 other *Mucor* species, *M*. *velutinosus* B5328 had the highest number of protein-coding genes in common with *M*. *circinelloides f*. *circinelloides* 1006PhL (7,522 orthologs) and *M*. *circinelloides* B8987 (7,140 orthologs) and the fewest with *M*. *irregularis* B50 (3,694 orthologs) (Fig 4A).[11] When comparing all 2,661 predicted orthologs shared by these 6 strains, *M*. *velutinosus* grouped most closely with *M*. *circincelloides* strains (Fig 4B). Compared to the other *Mucor* species, 1,354 open reading frames were unique to *M*. *velutinosus* B5328, including 66 putative genes predicted to encode secreted proteins, several of which are secreted aspartyl proteinases (SAPs), which are implicated in numerous functions during the infection process of fungi, such as *Candida albicans* (Table 2) [40, 41]. Consistent with the aggressive nature of our patient’s infection, strain RM1 was more virulent in a *Drosophila* challenge model compared to three clinical *M*. *circinelloides* isolates (Fig 4C), which was found to be the *Mucor spp* most closely related to *M*. *velutinosus* via genomic analyses.

**Table 1:** Basic Assembly and Structural Annotation Statistics for *M*. *velutinosus* B5328.

**Assembly statistics**	
Total scaffold length (Mb)	35.9
Average sequencing coverage	108x
G+C content	40.5%
Scaffolds	1,595
**Predicted protein coding genes**	
Predicted genes	9,855
Average coding sequence length (nt)	1,634
Average protein length (amino acids)	466
Average exons per mRNA	4.2
**Predicted non-coding RNA genes**	
Predicted genes	249

**Table 2:** Functional Annotation for 66 Secreted Proteins Unique to *M*. *velutinosus* B5328.

Annotation	Number
60S acidic ribosomal protein P2	1
Beta-14-mannosyl-glycoprotein 4-beta-N-acetylglucosaminyltransferase	1
Calcium-channel protein cch1	1
Cell wall protein PRY3	1
Disintegrin and metalloproteinase domain-containing protein B	1
Frizzled and smoothened-like protein G	1
Gastricsin (aspartyl proteinase)	3
Glucan 13-beta-glucosidase	1
hypothetical protein	44
Magnesium transporter NIPA2	1
Meiotic coiled-coil protein 2	1
Mucorpepsin (aspartyl proteinase)	1
Papain inhibitor	1
Probable glycosidase C21B10.07	1
Protein ERGIC-53	1
T-cell immunomodulatory protein	1
Tetratricopeptide repeat protein 7A	1
Uncharacterized amino-acid permease C15C4.04c	1
Uncharacterized protein YcaC	3

### Host Whole Exome Sequencing

The unusual timing of invasive mucormycosis, the rarity of the infecting *Mucor* species, and the history of recalcitrant onychomycosis led us to hypothesize that this uncommon clinical scenario could be attributed to patient polymorphisms in genes known to be or putatively involved in control of fungal pathogens. Given that there are minimal data regarding mucormycosis susceptibility [42], we performed an unbiased approach using whole exome sequencing of patient germline DNA with subsequent analysis of variation in all genes currently thought to contribute to immune function [22]. We detected six distinct polymorphisms that have been associated with susceptibility to fungal infection and/or alteration of innate immune function (Table 3). The patient was homozygous for both pentraxin 3 (*PTX3*) +743AA p.A48D allele (rs3816527) and c.530-8584T>C (rs2305619), which are in linkage disequilibrium and were recently characterized as significantly associated with increased incidence of invasive aspergillosis in hematopoietic stem-cell transplant (HSCT) recipients and invasive mold infections in solid organ transplant recipients [43, 44]. Similarly, the patient was heterozygous for the +745TC allele in the Toll-like receptor 6 (*TLR6*) gene which confers a p.S249P change (rs5743810) that has also been associated with invasive aspergillosis in HSCT recipients [45]. Other potentially significant polymorphisms in innate immune function genes included heterozygosity for *CARD15*/*NOD2* p.R702W change, *DDX58(RIG-I)* p.R7C substitution, and a frameshift-inducing deletion of 32 bps in *CCR5* (Table 3). NOD2 encodes an intracellular innate immunity sensor, and the *NOD2* p.R702W polymorphism is strongly associated with inflammatory bowel disease and bacterial infections [46]. DDX58 (RIG-I) also participates in host sensing of microbes, particularly viruses, and the mutation p.R7C has been shown to decreasethe innate immune response of dendritic cells [47]. CCR5 is present on the surface of T-cells, is involved in T-cell signaling in response to various microbes, and the 32 bp deletion in *CCR5* is most notably linked to resistance to HIV infection but has also been implicated in a variety of immune system functions [48]. Finally, the patient was homozygous for a stop-gain polymorphism p.W154X in the fucosyltransferase 2 (*FUT2*) gene, which leads to a fucose non-secretor phenotype, possibly impacting the patient’s microbiome as discussed below [49].

**Table 3:** Potential Host Polymorphisms Influencing *M*. *velutinosus* Infection Identified by Whole Exome Sequencing.

Gene	Allele frequency (depth)	dbSNP ID	Amino Acid change	Comments
***PTX3***	1.0 (45)	rs2305619	None (intronic)	Increased IA risk in HSCT recipients [43]
***PTX3***	1.0 (53)	rs3816527	p.A48D	See above
***TLR6***	0.48 (102)	rs5743810	p.S249P	Increased IA risk in HSCT recipients [45]
***NOD2***	0.47 (64)	rs2066844	p.R702W	Associated with Crohn’s disease [46]
***DDX58/RIG-I***	0.45 (29)	rs10813831	p.R7C	Decreased receptor function [47]
***CCR5***	0.29 (165)	None	p.184_194del	Resistance to HIV infection [48]
***FUT2***	0.99 (225)	rs6013338	p.W154X	Leads to non-secretor phenotype [49]

IA, invasive aspergillosis; HSCT, hematopoietic stem cell transplant

### Microbiome and Mycobiome Characterization

**Fig 5:**
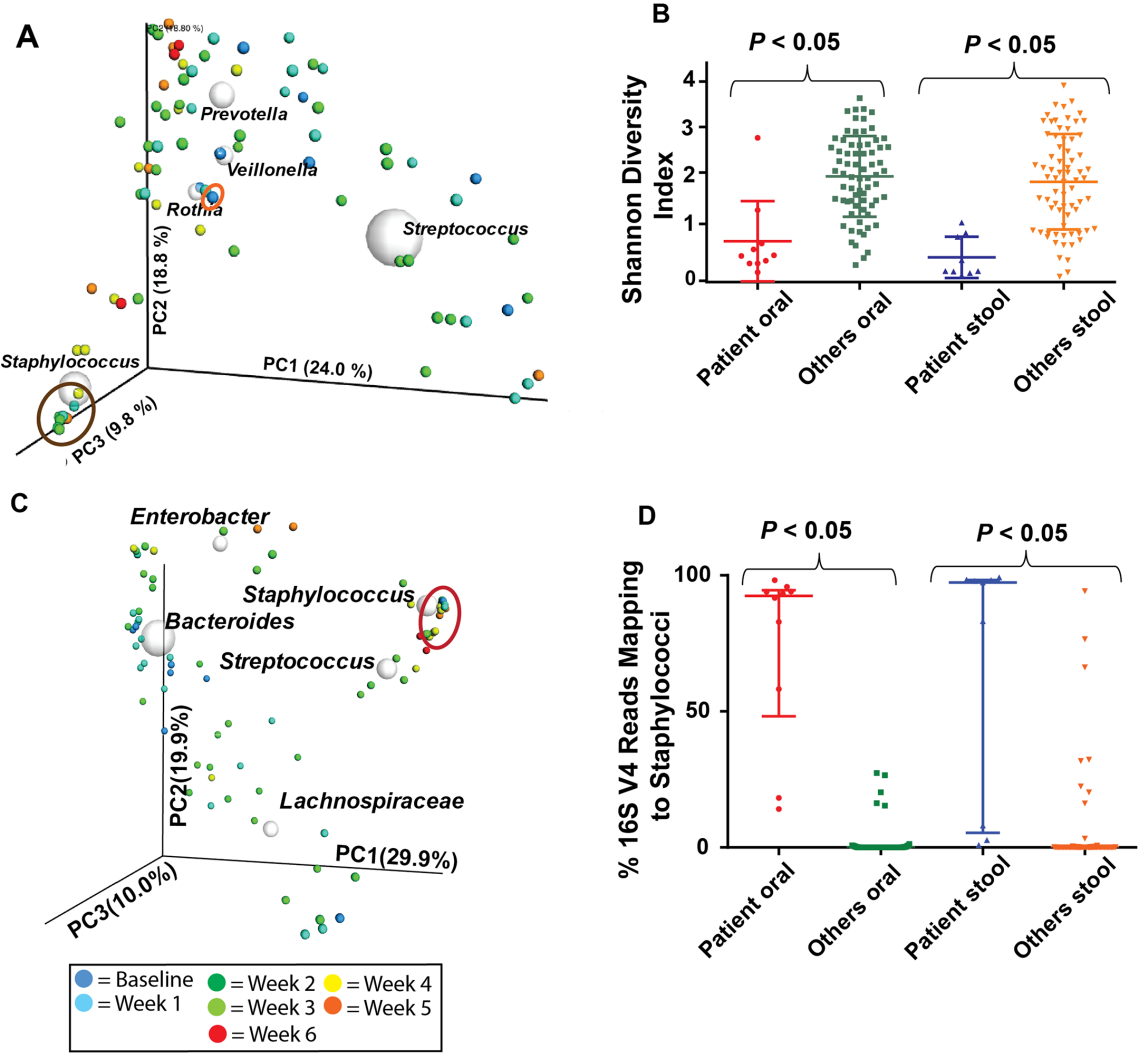
Characterization of Microbiome Leading up to Mucormycosis Onset. (A) Principal Coordinate Analysis (PCoA) of the phylogenetic differences observed in longitudinal oral samples (dominant genera identified plotted as white spheres). Buccal swabs were collected twice-weekly (color coding dating from onset of chemotherapy is shown in box in figure) from ten patients with acute myelogenous leukemia over the course of induction chemotherapy. Baseline sample from patient with *M*. *velutinosus* infection is circled in orange whereas subsequent samples were dominated by staphylococci, clustered together, and are circled in brown. Axes show largest amount of explained variation in the dataset with numbers referring to percent of variation explained by each axis. (B) Shannon diversity index of oral and stool samples from patient compared to the nine other sampled patients. Data graphed are mean ± standard deviation, and *P* values refer to Student’s *t*-test comparing the *M*. *velutinosus*-infected patient’s sample to samples from the other nine patients. (C) PCoA of the phylogenetic differences observed in longitudinal stool samples from ten patients as per panel (A). Samples from the patient with *M*. *velutinosus* infection were dominated by staphylococci, clustered together, and are circled in red. (D) % of all reads of V4 region of 16s RNA mapping to staphylococci with data divided as per panel (B). Data graphed are median ± interquartile range with *P* values referring to comparison of the *M*. *velutinosus* infected patient’s sample to samples from the other nine patients using the Mann-Whitney test as data were not normally distributed.

Although it has long been thought that host factors are paramount in controlling fungal proliferation, it is increasingly understood that commensal microorganisms also play a critical role in limiting the development of opportunistic fungal infections [50]. Thus, we next sought to determine the composition of the patient’s oral and stool microbiome leading up to disease onset. The patient’s initial oral microbiome was relatively diverse (Shannon index = 2.59, reads mapping to staphylococci = 19%), but within 3 days of starting AML therapy the oral microbiome exhibited a low diversity environment dominated by staphylococci (mean Shannon index of non-baseline samples = 0.48 ± 0.33, mean percent of reads mapping to staphylococci from non-baseline samples = 80% ± 27%) (Fig 5A and 5B). Similarly, the stool microbiome of the patient also had low microbial diversity (mean Shannon index = 0.39 ± 0.33) with a predominance of staphylococci (mean percent of reads mapping to staphylococci = 65% ± 46%) (Fig 5C and 5D). Such staphylococcal dominance was not characteristic of nine other patients with AML undergoing IRC, none of which developed mucormycosis (Fig 5).

**Fig 6:**
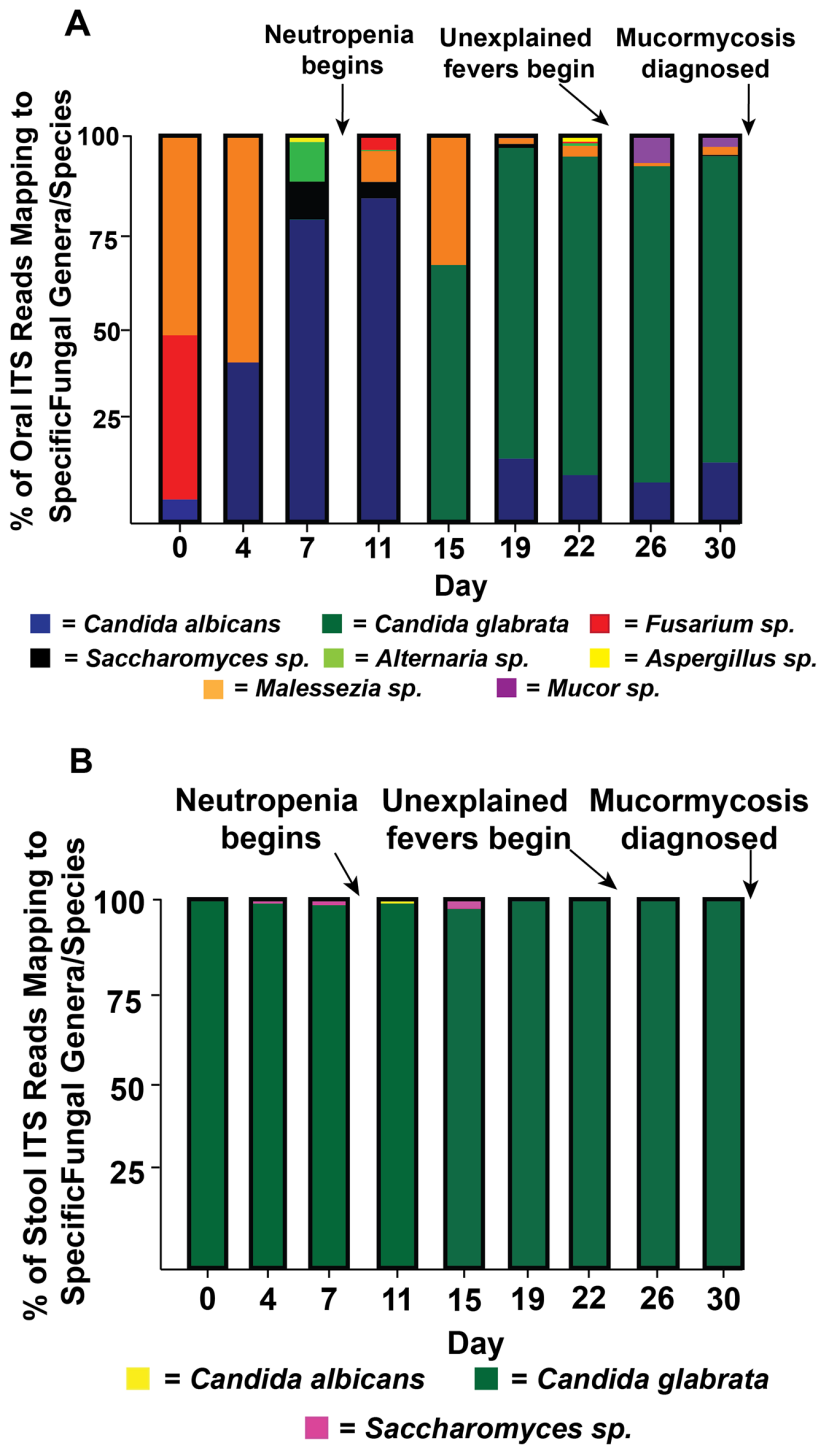
Characterization of Mycobiome Leading up to Mucromycosis Onset. (A) Longitudinal analysis of the patient’s oral mycobiome leading up to the onset of *M*. *velutinosus* infection. Buccal swabs were collected from the patient twice weekly and analyzed via sequencing of the internal transcribed spacer 2 (ITS2) region. Fungal genera/species that comprised at least 1% of mapped reads are depicted. (B) Data are as for panel A except that stool specimens were analyzed.

We sought to examine whether the low diversity microbiome could be correlated with *M*. *velutinosus* proliferation by determining the mycobiome of the patient from the same longitudinal oral and stool samples. Consistent with a previous report [51], several genera of potentially pathogenic fungi were detected in the oral samples including *Aspergillus* and *Fusarium* (Fig 6A). Intriguingly, *Mucor* was not initially detectable in the oral mycobiome, but its appearance coincided with the onset of unexplained fever which preceded the diagnosis of invasive mucormycosis (Fig 6A). *C*. *glabrata* dominated the stool mycobiome throughout the entire course of AML treatment accounting for >98% of reads at all time points measured, with no pathogenic molds being detected in any stool sample (Fig 6B).

## Discussion

The dramatic decrease in nucleotide sequencing costs has opened a new era for investigation of microbial pathogenesis [52]. In turn, genome-wide investigations targeting a specific aspect of the host-infecting microbe(s)-commensal microbiota paradigm have generated numerous novel and important insights into the pathogenesis of infectious diseases in humans [3, 4, 53]. However, the impactful nature of each approach underscores the idea that a more complete understanding of why particular humans develop disease and variance in clinical outcomes will require data incorporating host, pathogen, and microbiota factors [54]. Herein we took advantage of the unusual development of a severe infection in a patient that was being prospectively monitored in the hospital setting to demonstrate the feasibility and impact of a pan-genomic approach to studying an infectious disease occurrence.

From an infecting microbe standpoint, the advent of genetic approaches to identification of the agents of mucormycosis is beginning to reveal the tremendous genetic diversity inherent in organisms that cause this devastating disease, which casts doubt on the current uniform approach to diagnosis and treatment [55]. Our finding that the infecting *Mucor* isolate was a member of the *M*. *velutinosus* species was unexpected given that *M*. *velutinosus* is only a recently described species that is not currently thought to be a major cause of mucormycosis in humans [37, 38]. However, the unusual dissemination aspect of our case is consistent with the data present in other case reports which together show a striking predilection for *M*. *velutinosus* to be isolated from the bloodstream and/or to disseminate to the skin [37, 39, 55]. Using comparative genomics of multiple *Mucor* species, we were able to identify a limited number of proteins unique to *M*. *velutinosus* that may contribute to disease dissemination, including putative SAPs. SAPs have been most extensively recognized as virulence factors in *Candida* species because of their ability to degrade host proteins, contribute to host cell adherence, and facilitate endothelial cell penetration [41]. Moreover, our data suggest that correlations of careful clinical observations with genetic characterization of large cohorts of mucormycosis causing isolates may facilitate unraveling of mucormycosis pathogenesis.

Genetic factors have long been known to predispose patients to invasive fungal infections, such as defects in the neutrophils oxidative burst pathway that underlie chronic granulomatous disease [56]. More recently, there has been increasing appreciation of the role played by more subtle genetic alterations in predisposing patients to invasive fungal infection which only become clinically apparent during periods of iatrogenic immunosuppression [25]. For example, the +734 AA allele in *PTX3*, which was present in our patient, was found to predispose HSCT and solid organ transplant recipients to invasive aspergillosis [43, 44]. PTX3 is a soluble molecule that facilitates fungal opsonophagocytosis by polymorphonuclear and epithelial cells [43]. However, our identification of functionally significant polymorphisms in multiple genes contributing to innate immune function (Table 1) also demonstrates the need to expand understanding of how combinations of polymorphisms in key effectors of immune function, such as the *PTX3* and *TLR6* polymorphisms observed in our patient, influence infectious diseases susceptibility. To this end, non-biased, whole exome or even whole genome based studies of large cohorts of patients at risk of invasive fungal infection are needed to more fully understand genetic factors driving susceptibility to invasive fungal infection in immunocompromised patients.

In addition to identifying polymorphisms in genes traditionally considered important to control of fungal pathogens, our patient also had polymorphisms in genes that may influence disease presentation via effects on the microbiota. For example, in a mouse model, the same *FUT2* non-secretor polymorphism present in our patient was found to confer decreased resistance to intestinal colonization by pathogenic organisms during times of stress, perhaps because commensal microbiota use fucose shed from the gastrointestinal epithelium as a nutrient source [57]. Similarly, the NOD2 polymorphism present in our patient has been closely linked with microbiota alterations and the development of inflammatory bowel disease [58]. Consistent with these genetic polymorphisms, our patient had a severely dysbiotic oral and stool microbiome that was dominated by staphylococci, a pattern that was not observed in other patients being treated for AML. The clinical significance of gastrointestinal domination by staphylococci is not clear at present, but the temporal relationship between such dominance and the emergence of invasive mucormycosis is intriguing. The last several years have seen a dramatic increase in understanding the role of commensal bacteria in preventing disease due to pathogenic fungi [50, 59]. For example, it has recently been shown that *Lactobacillus* mediated production of IL-22 is critical to colonization resistance to *Candida albicans* [60]. Thus, we hypothesize that the dysbiotic nature of the oral microbiome may have provided a permissive environment for establishment and the eventual development of invasive mucormycosis which became detectable through genetic means some 7 days prior to the clinical diagnosis. These findings generate new interest in understanding mechanisms driving maintenance or loss of microbial diversity during cancer therapy or other times of physiologic stress encountered by humans during medical illness as well as the raise the possibility of using microbiome monitoring as a means for early detection of life-threatening fungal infections in immunocompromised hosts.

## Conclusions

Herein, we investigated invasive mucormycosis by applying a pan-genomic approach to infecting microbe-holobiont interactions. We identified contributions from host, infecting microbe, and commensal microorganisms that moved our understanding of this disease process far beyond what was possible through a standard clinical assessment. Importantly, these findings are best understood contextually and in combination, demonstrating the potential power such multifaceted methodology has to fully apply a concept of personalized medicine that includes the commensal microflora to infectious diseases pathogenesis. Further implementation of the tactics outlined herein could help generate new insights into how synergistic interactions driven by variations in host genetics, pathogenomics, and micro-/mycobiome composition combine to influence human disease.
